# Gene specific modifications unravel ethanol and acetaldehyde actions

**DOI:** 10.3389/fnbeh.2013.00080

**Published:** 2013-07-08

**Authors:** Yedy Israel, Mario Rivera-Meza, Eduardo Karahanian, María E. Quintanilla, Lutske Tampier, Paola Morales, Mario Herrera-Marschitz

**Affiliations:** ^1^Faculty of Medicine, Molecular and Clinical Pharmacology Program, University of ChileSantiago, Chile; ^2^Department of Pharmacological and Toxicological Chemistry, University of ChileSantiago, Chile; ^3^Faculty of Medicine, Center for Biomedical Research, Diego Portales UniversitySantiago, Chile

**Keywords:** alcohol dehydrogenase, catalase, aldehyde dehydrogenase, reinforcement, aversion

## Abstract

Ethanol is metabolized into acetaldehyde mainly by the action of alcohol dehydrogenase in the liver, while mainly by the action of catalase in the brain. Aldehyde dehydrogenase-2 metabolizes acetaldehyde into acetate in both organs. Gene specific modifications reviewed here show that an increased liver generation of acetaldehyde (by transduction of a gene coding for a high-activity liver alcohol dehydrogenase ADH1^*^B2) leads to increased blood acetaldehyde levels and aversion to ethanol in animals. Similarly aversive is an increased acetaldehyde level resulting from the inhibition of liver aldehyde dehydrogenase-2 (ALDH2) synthesis (by an antisense coding gene against *aldh2* mRNA). The situation is diametrically different when acetaldehyde is generated in the brain. When the brain ventral tegmental area (VTA) is endowed with an increased ability to generate acetaldehyde (by transfection of liver rADH) the reinforcing effects of ethanol are increased, while a highly specific inhibition of catalase synthesis (by transduction of a shRNA anti catalase mRNA) virtually abolishes the reinforcing effects of ethanol as seen by a complete abolition of ethanol intake in rats bred for generations as high ethanol drinkers. Data shows two divergent effects of increases in acetaldehyde generation: aversive in the periphery but reinforcing in the brain.

## Introduction: the ethanol molecule

Ethanol became part of our ecology over 200 million years ago when yeast started fermenting carbohydrates in fruits and grains, generating ethanol (Ratcliff et al., 2012). Animals were subsequently exposed to ethanol in these naturally fermented products. It has been proposed (Duddley, 2000; Dominy et al., 2004) that animals that perceived ethanol as a pleasant substance had an evolutionary advantage since they also increased the intake of calories from these sources.

The pleasant effects of alcohol explain why humans engaged in manufacturing it. The book of Anni, in 1700 BC Egypt describes an overt intoxication and the rules of proper behavior to be followed in a beer shop. Subsequently, its distillation is described in 900 AD and its production and massive consumption were brought by the Industrial Revolution (Hogarth, 1751). While in moderate doses alcohol generates motivational and reinforcing effects, in high doses it can generate aversion, inducing cognitive deficits, motor incoordination and emesis.

There is general agreement that in high concentrations ethanol modifies some neurotransmitter receptors by allosteric binding to their hydrophobic pockets, for example on the gamma-amino butyric acid receptor (GABA-A) (Harris and Allan, 1985; Suzdak et al., 1986; Huidobro-Toro et al., 1987). These hydrophobic pockets are also modifiable, with similar effects, by barbiturates and anesthetics, such as enfluorane, isofluorane and other long chain aliphatic alcohols (Levitan et al., 1988; Pritchett et al., 1989; Mihic et al., 1994, 1997; Krasowski et al., 1998).

Another receptor upon which ethanol generates hypnotic/anesthetic effects is the N-Methyl-D-Aspartate (NMDA) glutamate receptor (Lovinger et al., 1989; Weight et al., 1992). Ethanol binds allosterically to the NMDA receptor (Wright et al., 1996; Wirkner et al., 1999), an effect that leads to the interruption of cognitive processes, consolidation of memory and anesthesia (Robbins and Murphy, 2006). The hydrophobic pockets in the NMDA receptor can also be modified by anesthetics of diverse structures such as halothane, cyclopropane and xenon (Ogata et al., 2006). However, several studies indicate that the hypnotic and anesthetic effects of ethanol do not correlate with its rewarding properties (Riley et al., 1977; Daoust et al., 1987; Elmer et al., 1990).

## Acetaldehyde as a mediator of the effects of ethanol

Crabbe et al. (2006) reviewed the literature on the putative role of 93 genes likely involved in the effects of ethanol. Those involved with greater frequency were alcohol dehydrogenase and aldehyde dehydrogenase. The present review deals primarily with studies that show the effects generated by increasing or reducing acetaldehyde levels and/or the ability of the liver and brain to generate it. The studies were conducted in Wistar-derived rats selectively bred for over 80 generations, which led to two lines of rats: the UChA (abstainer) and the UChB (bibulous). The studies to be presented dovetail with many other studies described in this issue and potentiate the concept that acetaldehyde is a major contributor of the effects of ethanol, both aversive and rewarding.

**Figure 1:**
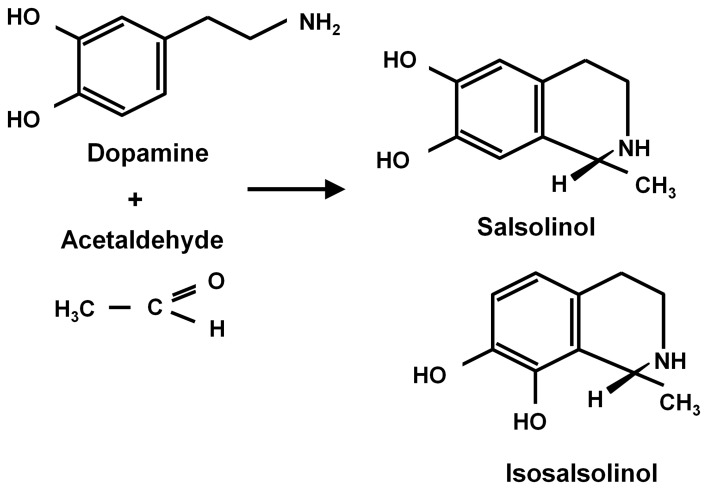
**Acetaldehyde condensation with dopamine.** Products formed in the non-enzymatic condensation are (R)- and (S)-salsolinol and (R)- and (S)- isosalsolinol (from Juricic et al., 2012).

Ethanol (CH3-CH2OH) is a small and relatively non-reactive molecule, thus requiring high concentrations to generate its effects. In high concentrations ethanol mainly interacts with hydrophobic pockets in proteins. On the other hand, acetaldehyde (CH3-CHO) is able to bind to amines (e.g., lysine residues) in proteins, generating Schiff bases (CH_3_-C=N-CH_2_-R), also binding to dopamine generating salsolinol, which has also been studied as a mediator of ethanol effects. Recently, Juricic et al. (2012) confirmed early studies by King et al. (1974) showing that acetaldehyde can, in addition to generating salsolinol, condense non-enzymatically with a carbon vicinal to a hydroxyl group in dopamine, yielding isosalsolinol (Figure 1). It was shown that commercial salsolinol (Sigma-Aldrich, sold prior to 2012) used in this field[,] is a mixture of four dopamine-acetaldehyde condensation products: *(R)- and (S)- salsolinol* (85%) and *(R)- and (S)- isosalsolinol* (10–15%).

## Aversive effects of liver-generated acetaldehyde

Among the first studies that described powerful effects of acetaldehyde were those seen in East Asians when consuming ethanol. Studies in the 80's demonstrated that 20–40% of individuals of East Asian origin (e.g., Japan, China, Korea) develop dysphoric effects when consuming alcohol; the effects often include peripheral vasodilatation and overt facial flushing, tachycardia, headache, nausea and emesis (Mizoi et al., 1983). Such individuals carry a point mutation in the gene that codes for the high affinity (low *K_m_*) mitochondrial aldehyde dehydrogenase-2 (*ALDH*2^*^2), a mutation that greatly reduces its affinity for NAD^+^, thus generating a virtually inactive dehydrogenase. Subjects carrying the *ALDH*2^*^2 polymorphism develop large increases in blood acetaldehyde (reaching 80–100 μM; over 5-fold that of subjects who carry the wild-type enzyme: *ALDH*2^*^1). It is important to note that these increases are due not only to the inability of liver to oxidize acetaldehyde into acetate, but also the inability of peripheral tissues and vascular tissues to oxidize acetaldehyde; tissues which also express the gene for high affinity mitochondrial aldehyde dehydrogenase. In venous blood of individuals carrying the normal ALDH2^*^1 enzyme, the levels of acetaldehyde are virtually nil; while levels of the order of 15–20 μM are found when arterial blood is sampled (see Quintanilla et al., 2007). The marked ability of endothelial tissues to metabolize acetaldehyde is of major importance in understanding why a lipophilic metabolite such as acetaldehyde does not cross the tight-junction cells that constitute the blood brain barrier (*vide infra*), and why the effects of acetaldehyde are so different in the periphery vs. the central nervous system.

A number of studies have shown that individuals carrying the *ALDH*2^*^2 allele are protected between 66% (heterozygous *ALDH*2^*^1/*ALDH*2^*^2) and 99% (homozygous *ALDH*2^*^2/*ALDH*2^*^2) against alcoholism (Harada et al., 1982; Thomasson et al., 1991; Higuchi, 1994; Tu and Israel, 1995; Chen et al., 1999; Luczak et al., 2006; Zintzaras et al., 2006). Hence, disulfiram (Antabuse®), a drug that non-specifically inhibits ALDH2, is the most efficient drug in the treatment of alcoholism provided its *daily intake* is secured by another person Chick et al. (1992), see meta-analysis by Jørgensen et al. (2011). Recent studies in animals (Escrig et al., 2012) show that disulfiram, while not showing behavioral effects on its own, reverses the anxiolytic effects of ethanol or shows anxiogenic effects. The administration of a large dose of acetaldehyde (100 mg/Kg) being anxiogenic *per se*.

Animal studies also support the concept that alcohol is a prodrug *vis-a vis* their aversive effects. Rats of the UChA line (virtually Abstainer) display a mutation in the *aldh2* gene (Sapag et al., 2003; see Quintanilla et al., 2006), which codes for an enzyme with a higher *K_m_* for NAD^+^ and a lower Vmax that the ALDH2 of heavy drinker animals (UChB; Bibulous). Further, the levels of arterial acetaldehyde display a large “acetaldehyde burst,” reaching 40–50 μM (vs. 10–20 μM for controls), which deters their alcohol intake (Quintanilla et al., 2005b). The mutation in the ALDH2 gene accounts for 50–60% of the low ethanol intake of UChA rats (Quintanilla et al., 2005a).

Additional evidence that systemic acetaldehyde is aversive was seen in gene modification studies that inhibited ALDH2 activity and elevated blood acetaldehyde levels. In studies by Ocaranza et al. (2008), UChB (drinker) rats were allowed access to 10% ethanol and water for 60 days (intakes of 7–8 g ethanol/kg/day), and were deprived of ethanol for 3 days. At the time of deprivation animals were injected an adenoviral vector (preferential liver tropism, but no crossing of blood-brain barrier) coding for an antisense RNA against ALDH2 mRNA, which lowered liver ALDH2 activity by 80–90% (*p* < 0.001). When ethanol access was re-allowed, control (empty virus) animals ingested 1.2 g ethanol/kg/60 min (10-fold higher than a naïve UChB) while animals treated with the anti ALDH2 antisense ingested 0.5–0.6 g ethanol/kg/60 min (*p* < 0.005). This inhibitory effect *remained constant* for the 34 days of the study. Acetaldehyde levels of animals that received the antisense against ALDH2 were of the order of 60 vs. 15–20 μM in controls.

In another study (Rivera-Meza et al., 2010), alcohol intake by UChB rats was reduced by 50% by the transfer into the liver (via an adenoviral vector) a rat homolog of the fast human alcohol dehydrogenase ADH1B^*^2 (ADH-47His) which elevated 6-fold liver ADH activity. In these studies, arterial acetaldehyde levels in the ADH1B^*^2 transduced animals increased from 20 to 80 μM after the i.p administration of 1.0 g ethanol/kg. These studies, in addition to confirming that increases in acetaldehyde at *physiological levels* generate alcohol aversion in the animals, explain a large number of studies showing that humans carrying the *ADH*1B^*^2 (*ADH-47His*) gene are protected against alcoholism (see meta-analysis by Zintzaras et al., 2006). The lack of understanding of why this enzyme protected against alcoholism stems from the fact that acetaldehyde in humans carrying the *ADH*1B^*^2 was determined in venous blood; being close to zero. As indicated earlier, venous blood, after having perfused the rich ALDH2^*^1 peripheral tissues are devoid of acetaldehyde.

From the above, genetic and pharmacokinetic evidence indicates that blood acetaldehyde in concentrations of 40–80 μM is aversive to animals and to humans. The mechanism by which ethanol generates vasodilatation, hypotension and nausea could be the release of histamine from mast cells (Shimoda et al., 1996). However, antihistaminic drugs do not increase the intake of ethanol in low drinker UChA rats (Quintanilla and Tampier, unpublished). We would like to point out that it is not clear if the aversive effects of acetaldehyde are generated in the periphery beyond the liver or actually start in nerves terminals that travel from the liver to the CNS, a matter that requires study.

## Brain acetaldehyde as a mediator of the rewarding effects of ethanol

**Figure 2:**
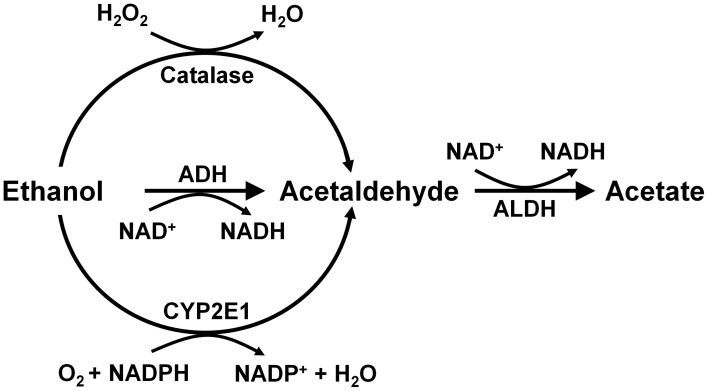
**Metabolism of ethanol into acetaldehyde by three enzymatic systems.** In the liver alcohol dehydrogenase (ADH) plays a major role, while catalase and cytochrome P450 (CYP2E1) play minor roles. In the brain, ADH (ADH1) is not expressed, but catalase mainly, and CYP2E1 to a minor extent, metabolize ethanol into acetaldehyde. Aldehyde dehydrogenase-2 (ALDH2) is present in virtually all cells (Drawn from Zimatkin et al., 2006).

Alcohol is absorbed from the stomach and intestine and is distributed throughout the body, reaching identical concentrations in the water of all tissues, including the brain. Ethanol is metabolized mainly in the liver by alcohol dehydrogenase (*K_m_* = 2 mM) generating acetaldehyde, which is further oxidized to acetate by a ubiquitous high affinity aldehyde dehydrogenase (*K_m_* < 0.2 μM). Other enzymes that oxidize ethanol into acetaldehyde are catalase and cytochrome p4502E1 (Figure 2). These latter enzymes play a minor role in metabolizing ethanol in the liver (Mezey, 1976; Khanna and Israel, 1980).

An important question in this field is whether liver-generated systemic acetaldehyde (normally not exceeding 20 μM in arterial blood after ethanol intake) can cross the blood brain barrier. Studies indicate that since the capillaries of the blood brain barrier have tight junctions (rather than open pores) acetaldehyde must first enter the ALDH2-rich endothelial cells of the barrier, which clear the acetaldehyde. Thus, under normal conditions of ethanol metabolism, systemic acetaldehyde does not cross the blood brain barrier (Eriksson, 1977; Lindros and Hillbom, 1979; Peterson and Tabakoff, 1979; Stowell et al., 1980). Only when systemic concentrations exceed 100 μM, following the administration of systemic acetaldehyde, acetaldehyde enters the CNS (Tabakoff et al., 1976). High concentrations of brain acetaldehyde can also be attained by the systemic (i.p. or s.c.) administration high doses of acetaldehyde.

Alcohol dehydrogenase is not expressed in the brain (see Zimatkin et al., 2006; Deitrich, 2011), however, acetaldehyde can be generated from ethanol by the catalase reaction, and to a minor extent by CYP2E1, both enzymes present in brain (Tampier and Mardones, 1979; Aragon et al., 1992; Gill et al., 1992; Zimatkin et al., 2006). *In vitro* studies indicate that catalase generates 60–70% of brain acetaldehyde while CYP2E1 some 20% (Zimatkin et al., 2006). *In vivo* studies by Zimatkin and Buben (2007) showed that ethanol infusion into the cerebral ventricles can generate acetaldehyde (achieving 60 μM), as detected in the cerebrospinal fluid. However, the concentrations of ethanol infused (85–90 mM) were in the anesthetic range (legal limits in most countries are 6–17 mM). In these studies, the catalase inhibitor aminotriazole reduced acetaldehyde levels in the cisterna magna. While such studies were promising in pointing out a major effect of catalase in acetaldehyde generation, the low specificity of aminotriazole required caution.

The question remains as to whether enough acetaldehyde is generated following a moderate ethanol intake to induce pharmacological effects. Studies in which aminotriazole was administered showed a reduction in voluntary ethanol intake by rats (Aragon and Amit, 1992; Tampier et al., 1995). However, aminotriazole also inhibited the consumption of food and of saccharine solutions (Rotzinger et al., 1994; Tampier et al., 1995), indicating non-specific actions. With a more direct approach, Ledesma and Aragon (2013) showed that reducing brain hydrogen peroxide levels (required by catalase to oxidize ethanol to acetaldehyde) reduced alcohol-induced conditioned place preference. Most early studies in the field have been conducted by either administering large doses of acetaldehyde or by the use of inhibitors or inducers of catalase. The reader is referred to recent reviews in this field (Quertemont et al., 2005; Deitrich, 2011; Correa et al., 2012). Overall, the field generally agrees with the view that acetaldehyde mediates the reinforcing effects of ethanol; however, the methodologies used to achieve such consensus are varied, in some cases employing non-physiological concentrations and routes of administration of ethanol or acetaldehyde or non-specific inhibitors.

Early studies showed that rats self-administer acetaldehyde intracerebrally (Amit et al., 1977; Brown et al., 1979; Amit and Smith, 1985), indicating a reinforcing effect of this metabolite at the central nervous system level. Rodd et al. (2005) demonstrated that rats selectively bred as alcohol drinkers (strain P of Indianapolis) self-administer both ethanol and acetaldehyde into the brain ventral tegmental area (VTA). Acetaldehyde (6 × 10^−6^ M) showed reinforcing effects at concentrations that were 1000 smaller than those required for ethanol (17 × 10^−3^ M) self-administration. The question remained as to whether enough acetaldehyde is generated in the brain to develop rewarding and reinforcing effects *when ethanol is consumed orally*.

**Figure 3:**
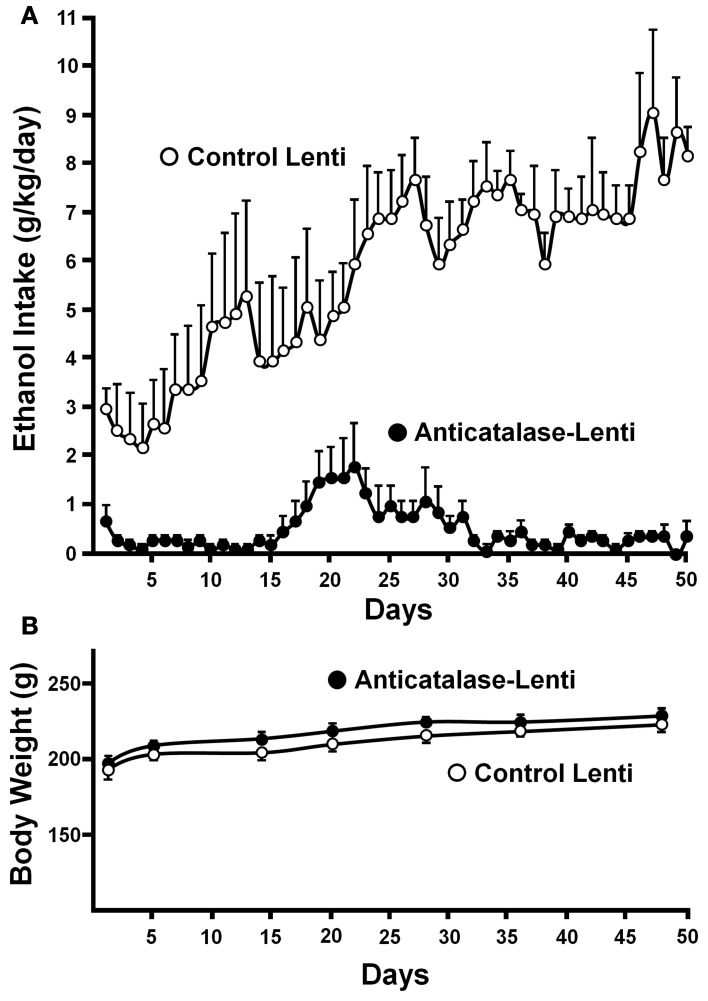
**Virtual long-lasting abolition of ethanol intake following the single administration of an anticatalase viral vector into the brain ventral tegmental area (VTA). (A)** Ethanol drinker rats (UChB line) were microinjected into the VTA 1.0 microliter (8 × 10^4^ particles) of a lentiviral vector coding for an shRNA against catalase mRNA. Controls received the empty lentiviral vector. Four days after the vector injection animals had 24-h access to 10% ethanol and water. **(B)** Animal weight was not affected by the anticatalase vector. Water intake (not shown) was not modified either (from Karahanian et al., 2011). Replicate studies by Quintanilla et al. (2012) showed that the anticatalase lentiviral vector reduced VTA catalase activity by 70–80%.

**Figure 4:**
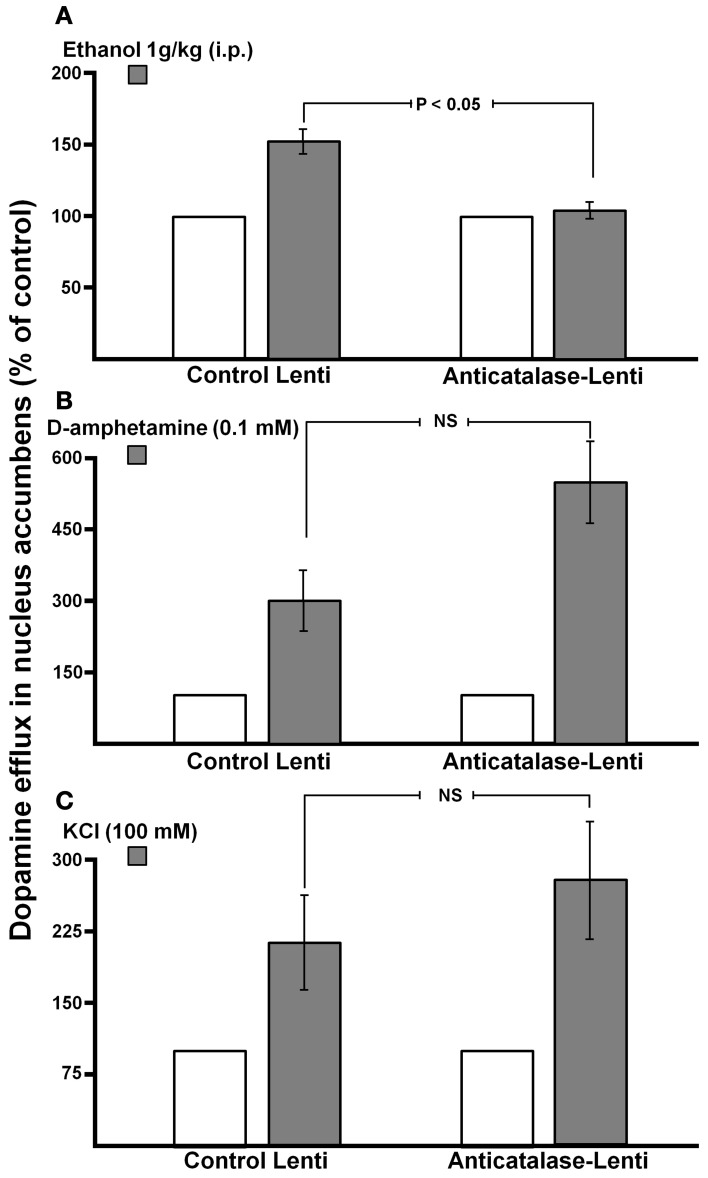
**Dopamine levels in the nucleus accumbens following acute ethanol administration.** Effect of anticatalase vector administration into the ventral tegmental area on dopamine release monitored in the ipsilateral nucleus accumbens. **(A)** Inhibition by anticatalase-lentiviral vector of dopamine efflux into the microdialysis fluid of nucleus accumbens (shell) induced by the systemic administration of ethanol (1 g/kg i.p.). **(B)** Anticatalase-lentiviral vector does not affect dopamine efflux into the microdialysis fluid of nucleus accumbens (shell) induced by d-amphetamine (0.1 mM) or **(C)** Anticatalase-lentiviral vector does not affect dopamine efflux into the microdialysis fluid of nucleus accumbens (shell) induced by KCl (100 mM) added to the microdialysis fluid. (From Karahanian et al., 2011).

**Figure 5:**
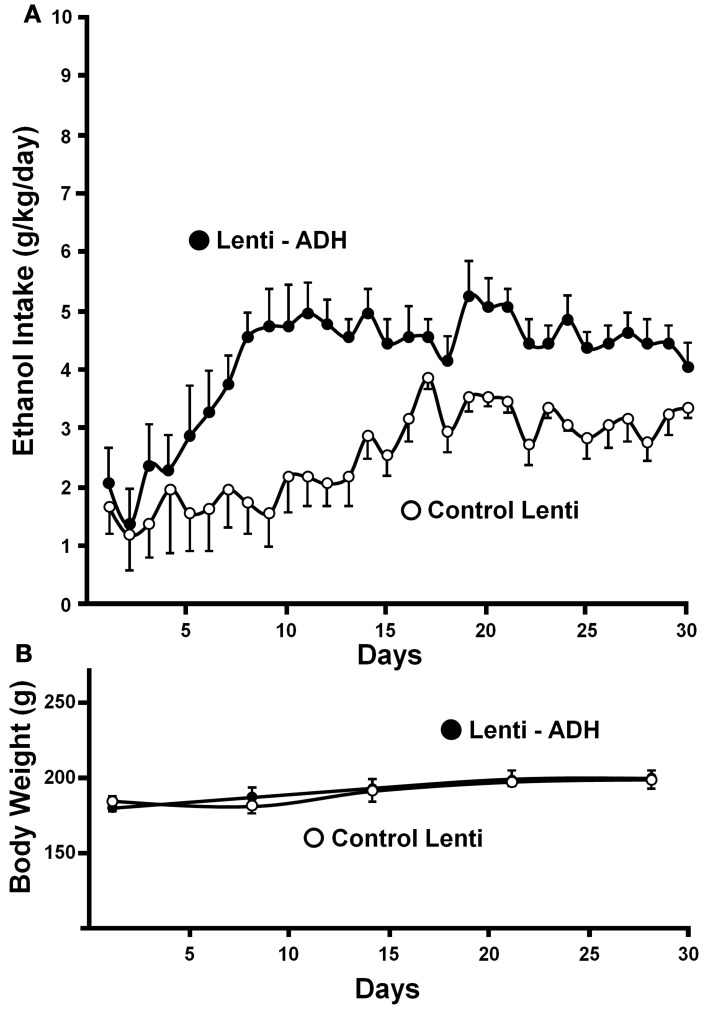
**Administration of the alcohol dehydrogenase coding gene (rat liver *adh*) into the VTA increases voluntary alcohol intake in rats (UChB) bred as alcohol drinkers. (A)** Four days after the injection of the lentiviral vectors, animals were allowed free availability of 5% (v/v) ethanol and water. Rats significantly (*p* < 0.001) increased their alcohol intake when injected with a lentiviral vector encoding alcohol dehydrogenase (rADH) (Lenti-ADH) into the ventral tegmental area compared to that observed after treatment with an empty lentiviral vector (control-Lenti) **(A)**. **(B)** No differences in body weight were observed along the experiment in Lenti-ADH-treated rats vs. control-Lenti virus-treated rats. Abscissa: days of ethanol availability. (From Karahanian et al., 2011).

Recently, a specific gene blocking technique allowed inhibiting brain catalase synthesis. Karahanian et al. (2011) developed lentiviral vectors coding a shRNA designed to inhibit the synthesis of catalase. Lentiviral vectors permanently integrate into the cell genome the genes they carry. A single stereotaxic administration of an anti-catalase lentiviral vector (anticatalase-lenti) into the VTA, which reduced catalase levels by 70–80% (Quintanilla et al., 2012), virtually abolished the voluntary ethanol consumption (up to 95%) by drinker UChB rats for 40–50 days (Karahanian et al., Figure 3). The lentiviral anticatalase shRNA administration also abolished the increased release of dopamine in nucleus accumbens induced by ethanol administration (Figure 4). It is noteworthy that rats were not unduly affected (water intake, body weight, behavior) by the administration of the anticatalase lentiviral vector (Karahanian et al., 2011), as in the brain enzymes other than catalase are mainly responsible for the elimination of hydrogen peroxide (Halliwell, 2006), namely glutathione peroxidases and most active peroxiredoxins (Turrens, 2003; Rhee et al., 2005). Overall, the rewarding effects of ethanol appear to be mediated by acetaldehyde generated in the brain by the action of catalase. One can hypothesize that an increased ability of VTA to generate acetaldehyde, induced by genetic modification, should also demonstrate an increased rewarding effect of ethanol. This was tested by the administration into the VTA of a lentiviral vector coding for liver alcohol dehydrogenase. In these studies, to avoid a ceiling of the rewarding effect of acetaldehyde generated by catalase (when 10% ethanol is available to the animals, ethanol intake approaches the rate of whole body degradation), animals were allowed access to 5% ethanol and water. As can be seen in Figure 5, animals transduced with the liver alcohol dehydrogenase (ADH) into the VTA significantly increased their ethanol intake compared to that of animals administered the control vector. The animals administered the vector coding liver ADH or the control vector did not show differences in body weight or behavior.

## Brain acetaldehyde as a mediator of the alcohol deprivation effect (ADE)

Sinclair and Senter (1968, 1977) showed that chronic intake of ethanol by rats, followed by a period of alcohol deprivation and subsequent re-exposure to ethanol leads animals to a marked increase of their ethanol intake above their basal pre-deprivation levels. This effect, termed the “*alcohol deprivation effect*” (ADE), is shown by a marked increase in voluntary intake of ethanol solutions (akin to binge-drinking) over baseline drinking when ethanol is reinstated after the period of alcohol deprivation (Spanagel and Hölter, 1999; Rodd-Henricks et al., 2001). An ADE can be observed after a short (1–3 days; Sinclair and Li, 1989; Agabio et al., 2000) or a long (up to 60–75 days) deprivation period (Sinclair et al., 1973; Spanagel and Hölter, 1999), but is not observed in non-deprived continuously alcohol-treated animals, suggesting that chronic exposure to ethanol alone is not sufficient to produce such a marked increase in ethanol intake (Spanagel and Hölter, 1999). Examination of the ADE phenomenon has revealed that at least 3–4 weeks of a continuous alcohol-drinking experience are required before deprivation to elicit an ADE (Spanagel and Hölter, 1999).

A number of studies showed that repeated alcohol intake–deprivation–re-administration episodes increase the expression of ADE (see Rodd et al., 2008) and Vengeliene and colleagues (2009) have shown that the motivational and reinforcing effects of ethanol are increased in the ADE condition, as animals experiencing the ADE will increase their work to procure ethanol.

**Figure 6:**
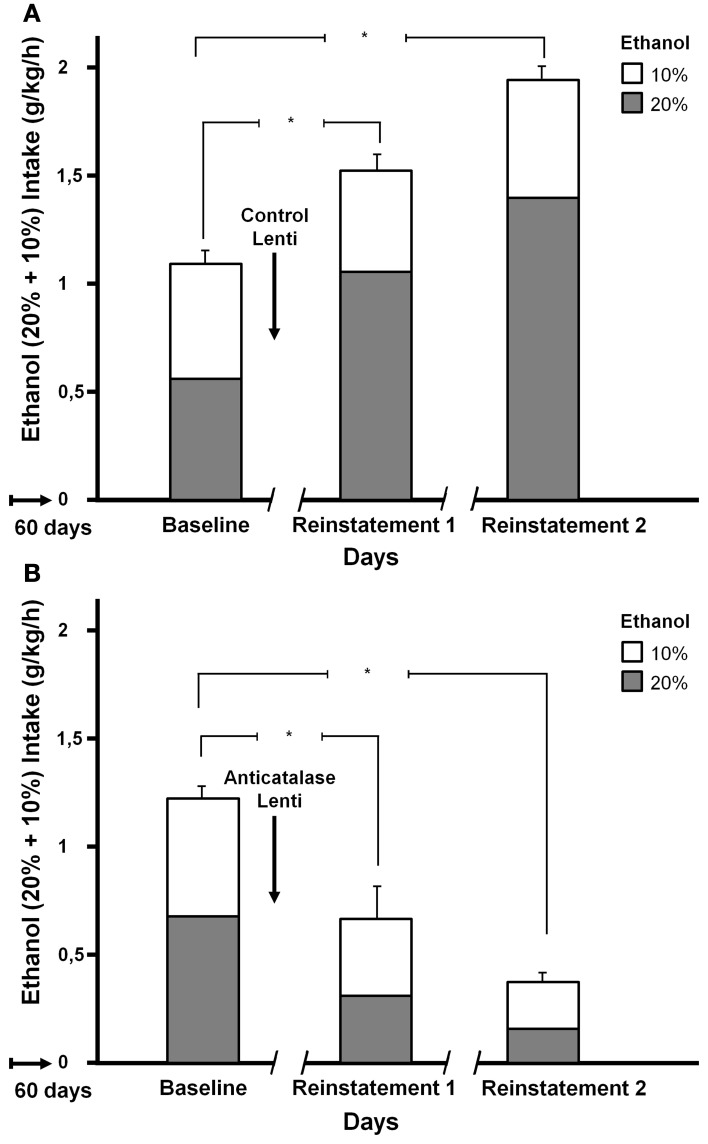
**Increased ethanol intake by UChB rats chronically exposed to ethanol, subsequently deprived and ethanol re-exposed.** Marked inhibition of intake following the administration of an anticatalase lentiviral vector. Animals with *ad-libitum* access to 10 and 20% ethanol and water for 60 days. Baseline data correspond to the average of ethanol intake restricted to only 1 h a day, for 7 days immediately prior to alcohol deprivation. A single intra-ventral tegmental area injection of an anticatalase-lentiviral vector inhibited the first 1-h ethanol intake after the first and second ethanol deprivation (ADE) periods of 15 days. The total height of each bar represents the sum of ethanol intake (g ethanol/kg/60 min) of the 10% solution (empty bars) plus that of the 20% solution (gray bars). The -/ /- symbol in the *x*-axis represents the 15-day deprivation period. **(A)** Control viral vector. **(B)** Anticatalase viral vector. Arrows indicate the administration of either control lentiviral vector or anticatalase-lentiviral vector prior to the 15 days of deprivation. The first and second re-exposure consumptions were significantly different from baseline. The inhibition induced by anticatalase vector administration was 67% (*p* < 0.001) after the first deprivation period, and 80% (*p* < 0.001) after the second deprivation period. Note also the marked increases in ethanol binge-drinking induced in control vector treated animals after the first and second ethanol intake and deprivation cycles (From Tampier et al., 2013).

Tampier et al. (2013) asked whether in the ADE condition a greater reinforcing effect of ethanol, leading to binge drinking, is also mediated by acetaldehyde (thus, also by products generated from acetaldehyde). It was postulated that if increases in ethanol intake induced by ADE were mediated by acetaldehyde, inhibition of VTA catalase synthesis by microinjection of an anticatalase lentiviral vector should inhibit ADE binge-drinking. To test this question rats were allowed for 60 days 24-h access to 10 and 20% ethanol solutions and water. On day 61, rats were divided into 2 groups matched for similar 24-h alcohol consumption and preference. One group received an intracerebral administration of the control lentiviral vector and was immediately deprived for 15 consecutive days of both the 10 and 20% ethanol solution, while water was the sole fluid available. The second group was injected into the VTA the anticatalase-Lenti-shRNA. As for the viral control group, these rats were returned to their home cage and deprived for 15 days of both 10 and 20% ethanol solutions. Following the 15 days of ethanol deprivation, re-exposure to free-choice intake of 10 and 20% ethanol and water started at 1 PM (on a normal daily cycle) and lasted for 7 days. Alcohol intake was recorded in all groups on the *first hour* of re-exposure (also for 24 h after alcohol re-exposure each day for 7 days; data not shown see Tampier et al., 2013). Thereafter, rats received a second period of 15 days of ethanol deprivation and further 7 days of ethanol drinking; again ethanol intake on the first hour of re-intake was recorded. Figure 6 indicates that (1) UChB rats reproduced the ADE binge drinking condition showing large amounts of ethanol consumed on the first hour post ethanol deprivation (achieving an intoxicating 2 g/kg/60 min) (2) subsequent deprivation ADE cycles increased ethanol intake with a greater consumption of the more concentrated ethanol solution (in line with a more reinforcing effect of ethanol after ADE), and (3) the increased in ethanol intake in the ADE condition was strongly inhibited by the anticatalase vector. After the second deprivation cycle ethanol intake was inhibited by 80%. The above study strongly suggests possible therapeutic avenues in the treatment of alcoholism. It should be noted that administration of viral vectors is used in human therapies (Kaplitt et al., 2007) and are approved by agencies such as the U.S. Food and Drug Administration (FDA). Additional studies are being conducted (Karahanian et al. under review) to determine if an overexpression of rat ALDH2 in the VTA also inhibits the ADE-induced increases in ethanol intake.

## Conclusions

Gene-based specific modifications show that an increased liver generation of acetaldehyde, leading to increased blood acetaldehyde levels, results in aversion to ethanol in animals. Similarly, aversion to ethanol results from an increased acetaldehyde level resulting from the inhibition of liver aldehyde dehydrogenase-2 synthesis. The situation is radically different when acetaldehyde is generated in the brain. When the brain ventral tegmental area is endowed with an increased ability to generate acetaldehyde the reinforcing effects of ethanol are increased, while a highly specific inhibition of catalase synthesis virtually abolishes the reinforcing effects of ethanol as seen by a complete abolition of ethanol intake. Data show two divergent effects of increases in acetaldehyde generation: aversive in the periphery but reinforcing in the brain.

### Conflict of interest statement

The authors declare that the research was conducted in the absence of any commercial or financial relationships that could be construed as a potential conflict of interest.

## References

[B1] Agabio R. Carais M. A. M. Lobina C. Pani M. Reai R. Vacca G. (2000). Development of short-lasting alcohol deprivation effect in Sardinian alcohol-preferring rat. Alcohol 21, 59–62 10.1016/S0741-8329(00)00072-0 10946158

[B2] Amit Z. Brown Z. W. Rockman G. E. (1977). Possible involvement of acetaldehyde, norepinephrine and their tetrahydroisoquinoline derivatives in the regulation of ethanol seld-administration. Drug. Alcohol Depend. 2, 495–500 10.1016/0376-8716(77)90049-7 913244

[B3] Amit Z. Smith B. R. (1985). A multi-dimensional examination of the positive reinforcing properties of acetaldehyde. Alcohol 2, 367–370 10.1016/0741-8329(85)90077-1 3893469

[B4] Aragon C. M. Amit Z. (1992). The effect of 3-amino-1 2, 4-triazole on voluntary ethanol consumption: evidence for brain catalase involvement in the mechanism of action. Neuropharmacology 31, 709–712 10.1016/0028-3908(92)90150-N 1407407

[B5] Aragon C. M. Rogan F. Amit Z. (1992). Ethanol metabolism in rat brain homogenates by a catalase-H2O2 system. Biochem. Pharmacol. 44, 93–98 10.1016/0006-2952(92)90042-H 1632841

[B7] Brown Z. W. Amit Z. Rockman G. E. (1979). Intraventricular self-administration of acetaldehyde, but not ethanol, in naive laboratory rats. Psychopharmacology 64, 271–276 10.1007/BF00427509 41277

[B8] Chen Y. C. Lu R. B. Peng G. S. Wang M. F. Wang H. K. Ko H. C. (1999). Alcohol metabolism and cardiovascular response in an alcoholic patient homozygous for the ALDH2*2 variant gene allele. Alcohol. Clin. Exp. Res 23, 1853–1860 10.1111/j.1530-0277.1999.tb04083.x 10630602

[B9] Chick J. Gough K. Kershaw P. Hore B. Mehta B. Ritson B. (1992). Disulfiram treatment of alcoholism. Br. J. Psychiatry 161, 84–89 10.1192/bjp.161.1.84 1638335

[B10] Correa M. Salamone J. D. Segovia K. N. Pardo M. Longoni R. Spina L. (2012). Piecing together the puzzle of acetaldehyde as a neuroactive agent. Neurosci. Biobehav. Rev, 36, 404–430 10.1016/j.neubiorev.2011.07.009 21824493

[B11] Crabbe J. C. Phillips T. J. Harris R. A. Arends M. A. Koob G. F. (2006). Alcohol-related genes: contributions from studies with genetically engineered mice. Addiction Biol. 11, 195–269 10.1111/j.1369-1600.2006.00038.x 16961758

[B12] Daoust M. Lhuintre J. P. Moore N. Saligaut C. Flipo J. L. Boismare F. (1987). Is initial sensitivity to ethanol correlated with alcohol preference in alcohol-drinking and non-drinking rats. Alcohol Alcohol. 22, 409–414 3426770

[B13] Deitrich R. (2011). Ethanol as a Prodrug: brain metabolism of ethanolmediates its reinforcing effects – a commentary. Alcohol. Clin. Exp. Res.35, 581–583 10.1111/j.1530-0277.2011.01454.x 21352247

[B14] Dominy N. J. Ross C. F. Smith T. D. (2004). Evolution of the special senses in primates: past, present, and future. Anat. Rec. A Discov. Mol. Cell. Evol. Biol. 281, 1078–1082 10.1002/ar.a.20112 15470667

[B15] Duddley T. (2000). Evolutionary origins of human alcoholism in primate frugivory. Q. Rev. Biol. 75, 3–15 10.1086/393255 10721531

[B16] Elmer G. I. Meish R. A. Goldberg S. R. George F. R. (1990). Ethanol self-administration in long sleep and short sleep mice indicates reinforcement is not inversely related to neurosensitivity. J. Pharmacol. Exp. Ther. 254, 1054–1062 2395106

[B17] Eriksson C. J. (1977). Acetaldehyde metabolism *in vivo* during ethanol oxidation. Adv. Exp. Med. Biol. 85A, 319–341 335831 10.1007/978-1-4899-5181-6_21

[B18] Escrig M. A. Pardo M. Aragon C. M. Correa M. (2012). Anxiogenic and stress-inducing effects of peripherally administered acetaldehyde in mice: similarities with the disulfiram-ethanol reaction. Pharmacol. Biochem. Behav. 100, 404–412 10.1016/j.pbb.2011.10.002 22005600

[B19] Gill K. Menez J. F. Lucas D. Deitrich R. A. (1992). Enzymatic production of acetaldehyde from ethanol in rat brain tissue. Alcohol. Clin. Exp. Res. 16, 910–915 10.1111/j.1530-0277.1992.tb01892.x 1443429

[B20] Halliwell B. (2006). Oxidative stress and neurodegeneration: where are we now. J. Neurochem. 97, 1634–1658 10.1111/j.1471-4159.2006.03907.x 16805774

[B21] Harada S. Agarwal D. P. Goedde H. W. Tagaki S. Ishikawa B. (1982). Possible protective role against alcoholism for aldehyde dehydrogenase isozyme deficiency in Japan. Lancet 2, 827 10.1016/S0140-6736(82)92722-2 6126701

[B22] Harris R. A. Allan A. M. (1985). Functional coupling of gamma-aminobutyric acid receptors to chloride channels in brain membranes. Science 228, 1108–1110 10.1126/science.2581319 2581319

[B23] Higuchi S. (1994). Polymorphisms of ethanol metabolizing enzyme genes and alcoholism. Alcohol Alcohol. Suppl 2, 29–34 8974313

[B24] Hogarth W. (1751). Gin lane, in Hogarth's Graphic Works, in Ronald Paulson Yale University Press, 1965.

[B25] Huidobro-Toro J. P. Bleck V. Allan A. M. Harris R. A. (1987). Neurochemical actions of anesthetic drugs on the gamma-aminobutyric acid receptor-chloride channel complex. J. Pharmacol. Exp. Ther. 242, 963–969 2443645

[B26] Jørgensen C. H. Pedersen B. Tønnesen H. (2011). The efficacy of disulfiram for the treatment of alcohol use disorder. Alcohol. Clin. Exp. Res. 35, 1749–1758 10.1111/j.1530-0277.2011.01523.x 21615426

[B27] Juricic M. A. Berríos-Cárcamo P. A. Acevedo M. L. Israel Y. Almodóvar I. Cassels B. K. (2012). Salsolinol and isosalsolinol: condensation products of acetaldehyde and dopamine. Separation of their enantiomers in the presence of a large excess of dopamine. J. Pharm. Biomed. Anal. 63, 170–174 10.1016/j.jpba.2012.02.002 22370127

[B28] Kaplitt M. G. Feigin A. Tang C. Fitzsimons H. L. Mattis P. Lawlor P. A. (2007). Safety and tolerability of gene therapy with an adeno-associated virus (AAV) borne GAD gene for Parkinson's disease: an open label, phase I trial. Lancet 369, 2097–2105 10.1016/S0140-6736(07)60982-9 17586305

[B29] Karahanian E. Quintanilla M. E. Tampier L. Rivera-Meza M. Bustamante D. Gonzalez-Lira V. (2011). Ethanol as a Prodrug: brain metabolism of etanol mediates its reinforcing effects. Alcohol. Clin. Exp. Res. 35, 606–612 10.1111/j.1530-0277.2011.01439.x 21332529 PMC3142559

[B30] Khanna J. M. Israel Y. (1980). Ethanol metabolism. Int. Rev. Physiol. 21, 275–315 6993397

[B31] King G. S. Goodwin B. L. Sandler M. (1974). Isosalsolinol formation: a secondary reaction in the Pictet-Spengler condensation, J. Pharm. Pharmacol. 26, 476–478 10.1111/j.2042-7158.1974.tb09323.x 4155002

[B32] Krasowski M. D. Koltchine V. V. Rick C. E. Ye Q. Finn S. E. Harrison N. L. (1998). Propofol and other intravenous anesthetics have sites of action on the gamma-aminobutyric acid type A receptor distinct from that for isoflurane. Mol. Pharmacol. 53, 530–538 9495821 10.1124/mol.53.3.530

[B33] Ledesma J. C. Aragon C. M. (2013). Acquisition and reconditioning of ethanol-induced conditioned place preference in mice is blocked by the H2O2 scavenger alpha lipoic acid. Psychopharmacology 226, 673–685 10.1007/s00213-012-2831-9 22885873

[B34] Levitan E. S. Schofield P. R. Burt D. R. Rhee L. M. Wisden W. Köhler M. (1988). Structural and functional basis for GABAA receptor heterogeneity. Nature 335, 76–79 10.1038/335076a0 2842688

[B35] Lindros K. O. Hillbom M. E. (1979). Acetaldehyde in cerebrospinal fluid: its near-absence in ethanol-intoxicated alcoholics. Med. Biol. 57, 246–247 513881

[B36] Lovinger D. M. White G. Wright F. F. (1989). Ethanol inhibits NMDA-activated ion current in hippocampal neurons. Science 243, 1721–1724 10.1126/science.2467382 2467382

[B37] Luczak S. E. Glatt S. J. Wall T. L. (2006). Meta-analyses of ALDH2 and ADH1B with alcohol dependence in Asians. Psychol. Bull. 132, 607–621 10.1037/0033-2909.132.4.607 16822169

[B38] Mezey E. (1976). Ethanol metabolism and ethanol-drug interactions. Biochem. Pharmacol. 25, 869–875 10.1016/0006-2952(76)90305-1 773384

[B39] Mihic S. J. McQuilkin S. J. Eger E. I. 2nd Ionescu P. Harris R. A. (1994). Potentiation of gamma-aminobutyric acid type A receptor-mediated chloride currents by novel halogenated compounds correlates with their abilities to induce general anesthesia. Mol. Pharmacol. 46, 851–857 7969071

[B40] Mihic S. J. Ye Q. Wick M. J. Koltchine V. V. Krasowski M. D. Finn S. E. (1997). Sites of alcohol and volatile anaesthetic action on GABA(A) and glycine receptors. Nature 389, 385–389 10.1038/38738 9311780

[B41] Mizoi Y. Tatsuno Y. Adachi J. Kigame M. Fukunaga T. Hishida S. (1983). Alcohol sensitivity related to polymorphism of alcohol-metabolizing enzymes in Japanese. Pharmacol. Biochem. Behav. 18(Suppl. 1), 127–133 10.1016/0091-3057(83)90159-4 6356156

[B42] Ocaranza P. Quintanilla M. E. Tampier L. Karahanian E. Sapag A. Israel Y. (2008). Gene therapy reduces ethanol intake in an animal model of alcohol dependence. Alcohol. Clin. Exp. Res. 32, 52–57 10.1111/j.1530-0277.2007.00553.x 18070247

[B43] Ogata J. Shiraishi M. Namba T. Smothers C. T. Woodward J. J. Harris R. A. (2006). Effects of anesthetics on mutant N-methyl-D-aspartate receptors expressed in Xenopus oocytes. J. Pharmacol. Exp. Ther. 318, 434–443 10.1124/jpet.106.101691 16622040

[B44] Oster S. M. Toalston J. E. Kuc K. A. Pommer T. J. Murphy J. M. Lumeng L. (2006). Effect of multiple alcohol deprivations on operant ethanol self-administration by high-ethanol drinking replicate rat lines. Alcohol 38, 155–164 10.1016/j.alcohol.2006.06.001 16905441

[B45] Peterson D. R. Tabakoff B. (1979). Characterization of brain acetaldehyde oxidizing systems in the mouse. Drug Alcohol Depend. 4, 137–144 10.1016/0376-8716(79)90054-1 510163

[B46] Pritchett D. B. Lüddens H. Seeburg P. H. (1989). Type I and type II GABAA-benzodiazepine receptors produced in transfected cells. Science 245, 1389–1392 10.1126/science.2551039 2551039

[B47] Quertemont E. Tambour S. Tirelli E. (2005). The role of acetaldehyde in the neurobehavioral effects of ethanol: a comprehensive review of animal studies. Prog. Neurobiol. 75, 247–274 10.1016/j.pneurobio.2005.03.003 15882776

[B48] Quintanilla M. E. Israel Y. Sapag A. Tampier L. (2006). The UChA and UChB rat lines: metabolic and genetic differences influencing ethanol intake. Addiction Biol. 11, 310–323 10.1111/j.1369-1600.2006.00030.x 16961761

[B49] Quintanilla M. E. Tampier L. Karahanian E. Rivera-Meza M. Herrera-Marschitz M. Israel Y. (2012). Reward and relapse: complete gene-induced dissociation in an animal model of alcohol dependence. Alcohol. Clin. Exp. Res. 36, 517–522 10.1111/j.1530-0277.2011.01606.x 21895710 PMC5958906

[B50] Quintanilla M. E. Tampier L. Sapag A. Gerdzen Z. Israel Y. (2007). Sex differences, alcohol dehydrogenase, acetaldehyde burst, and aversion to ethanol in the rat: a systems perspective. Am. J. Physiol. Endocrinol. Metab. 293, E531–E537 10.1152/ajpendo.00187.2007 17488809

[B51] Quintanilla M. E. Tampier L. Valle-Prieto A. Sapag A. Israel Y. (2005a). Complex I regulates mutant mitochondrial aldehyde dehydrogenase activity and voluntary ethanol consumption in rats. FASEB J. 19, 36–42 10.1096/fj.04-2172com 15629893

[B52] Quintanilla M. E. Tampier L. Sapag. Israel Y. (2005b). Polymorphisms in the mitochondrial aldehyde dehydrogenase gene (Aldh2) determine peak blood acetaldehyde levels and voluntary ethanol consumption in rats. Pharmacogenet. Genomics 15, 427–431 10.1097/01213011-200506000-00009 15900217

[B53] Ratcliff W. C. Denison R. F. Borrelo M. Travisano M. (2012). Experimental evolution of multicellularity. Proc. Natl. Acad. Sci. U.S.A. 109, 1595–1600 10.1073/pnas.1115323109 22307617 PMC3277146

[B54] Rhee S. G. Chae H. Z. Kim K. (2005). Peroxiredoxins: a historical overview and speculative preview of novel mechanisms and emerging concepts in cell signaling. Free Radic. Biol. Med. 38, 1543–1552 10.1016/j.freeradbiomed.2005.02.026 15917183

[B55] Riley E. P. Worsham E. D. Lester D. Freed E. X. (1977). Selective breeding of rats for differences in reactivity to alcohol. An approach to an animal model of alcoholism. II. Behavioral measures. J. Stud. Alcohol 38, 1705–1717 562458 10.15288/jsa.1977.38.1705

[B56] Rivera-Meza M. Quintanilla M. E. Tampier L. Mura C. V. Sapag A. Israel Y. (2010). Mechanism of protection against alcoholism by an alcohol dehydrogenase polymorphism: development of an animal model. FASEB J. 24, 266–274 10.1096/fj.09-132563 19710201 PMC2797030

[B57] Robbins T. W. Murphy E. R. (2006). Behavioural pharmacology: 40+ years of progress, with a focus on glutamate receptors and cognition. Trends Pharmacol. Sci. 27, 141–148 10.1016/j.tips.2006.01.009 16490260 PMC1867319

[B58] Rodd Z. A. Bell R. L. Kuc K. A. Murphy J. M. Lumeng L. McBride W. J. (2008). Effects of concurrent access to multiple ethanol concentrations and repeated deprivations on alcohol intake of high-alcohol-drinking (HAD) rats. Addiction Biol. 14, 152–164 10.1111/j.1369-1600.2008.00140.x 19076927 PMC2858401

[B59] Rodd Z. A. Bell R. L. Zhang Y. Murphy J. M. Goldstain A. Zafarroni A. (2005). Regional heterogeneity for the intracranial self-administration of ethanol and acetaldehyde within the ventral tegmental area of alcohol-preferring (P) rats: involvement of dopamine and serotonin. Neuropsychopharmacology 30, 330–338 10.1038/sj.npp.1300561 15383830

[B60] Rodd-Henricks Z. A. Bell R. L. Kuc K. A. Murphy J. M. McBride W. J. Lumeng L. (2001). Effect of concurrent access to multiple ethanol concentrations and repeated deprivations on alcohol intake of alcohol-preferring rats. Alcohol. Clin. Exp. Res. 25, 1140–1150 10.1111/j.1530-0277.2001.tb02328.x 11505045

[B61] Rotzinger S. Smith B. R. Amit Z. (1994). Catalase inhibition attenuates the acquisition of ethanol and saccharin-quinine consumption in laboratory rats. Behav. Pharmacol. 5, 203–209 10.1097/00008877-199404000-00012 11224269

[B62] Sapag A. Tampier L. Valle-Prieto A. Quintanilla M. E. Moncada M. E. Israel Y. (2003). Mutations in mitochondrial aldehyde dehydrogenase (ALDH2) change cofactor affinity and segregate with voluntary alcohol consumption in rats. Pharmacogenetics 13, 509–515 10.1097/00008571-200308000-00009 12893989

[B63] Shimoda T. Kohno S. Takao A. Fujiwara C. Matsuse H. Sakai H. (1996). Investigation of the mechanism of alcohol-induced bronchial asthma. J. Allergy Clin. Immuno. 97, 74–84 10.1016/S0091-6749(96)70285-3 8568140

[B64] Sinclair J. D. Li T.-K. (1989). Long and short alcohol deprivation effects on AA and P alcohol-preferring rats. Alcohol 6, 505–509 10.1016/0741-8329(89)90059-1 2597353

[B65] Sinclair J. D. Senter R. J. (1968). Development of an alcohol-deprivation effect in rats. Q. J. Stud. Alcohol 29, 863–867 5705408

[B66] Sinclair J. D. Senter R. J. (1977). Increased preference for ethanol in rats following deprivation Psychon. Sci. 8, 11–12

[B67] Sinclair J. D. Walker S. Jordan W. (1973). Behavioral and physiological changes associated with various durations of alcohol deprivation in rats. Q. J. Stud. Alcohol 34, 544–757 4795453

[B68] Spanagel R. Hölter S. M. (1999). Long-term alcohol self-administration with repeated alcohol deprivation phases: an animal model of alcoholism. Alcohol Alcohol. 34, 231–243 10.1093/alcalc/34.2.231 10344783

[B69] Stowell A. Hillbom M. Salaspuro M. Lindros K. O. (1980). Low acetaldehyde levels in blood, breath and cerebrospinal fluid of intoxicated humans as assayed by improved methods. Adv. Exp. Med. Biol. 132, 635–645 7424734 10.1007/978-1-4757-1419-7_66

[B70] Suzdak P. D. Glowa J. R. Crawley J. N. Schwartz R. D. Skolnick P. Paul S. M. (1986). A selective imidazobenzodiazepine antagonist of ethanol in the rat. Science 234, 1243–1247 10.1126/science.3022383 3022383

[B71] Tabakoff B. Anderson R, A. Ritzmann R. F. (1976). Brain acetaldehyde after ethanol administration. Biochem. Pharmacol. 25, 1305–1309 10.1016/0006-2952(76)90094-0 938553

[B72] Tampier L. Mardones J. (1979). Catalase mediated oxidation of ethanol by rat brain homogenates. IRCS Med. Sci. 7, 389

[B73] Tampier L. Quintanilla M. E. Karahanian E. Rivera-Meza M. Herrera-Marschitz M. Israel Y. (2013). The alcohol deprivation effect:marked inhibition by anticatalase gene administration into the ventral tegmental area in rats Alcohol Clin. Exp. Res. [Epub ahead of print]. 10.1111/acer.12101 23527889

[B74] Tampier L. Quintanilla M. E. Mardones J. (1995). Effects of aminotriazole on ethanol, water, and food intake and on brain catalase in UChA and UChB rats. Alcohol 12, 341–344 10.1016/0741-8329(95)00014-I 7546330

[B75] Thomasson H. R. Edenberg H. J. Crabb D. W. Mai X. L. Jerome R. E. Li T. K. (1991). Alcohol and aldehyde dehydrogenase genotypes and alcoholism in Chinese men. Am. J. Hum. Genet. 48, 677–681 2014795 PMC1682953

[B76] Tu G. C. Israel Y. (1995). Alcohol consumption by Orientals in North America is predicted largely by a single gene. Behav. Genet. 25, 59–65 10.1007/BF02197242 7755519

[B77] Turrens J. F. (2003). Mitochondrial formation of reactive oxygen species. J. Physiol. 552, 335–344 10.1113/jphysiol.2003.049478 14561818 PMC2343396

[B78] Vengeliene V. Celerier E. Chaskiel L. Penzo F. Spanagel R. (2009). Compulsive alcohol drinking in rodents. Addiction Biol. 14, 384–396 10.1111/j.1369-1600.2009.00177.x 19740366

[B79] Weight F. F. Aguayo L. G. White G. Lovinger D. M. Peoples R. W. (1992). GABA- and glutamate-gated ion channels as molecular sites of alcohol and anesthetic action. Adv. Biochem. Psychopharmacol. 47, 335–347 1354918

[B80] Wirkner K. Poelchen W. Köles L. Múlberg K. Scheibler P. Allgaier C. (1999). Ethanol-induced inhibition of NMDA receptor channels. Neurochem. Int. 35, 153–162 10.1016/S0197-0186(99)00057-1 10405999

[B81] Wright J. M. Peoples R. W. Weight F. F. (1996). Single-channel and whole-cell analysis of ethanol inhibition of NMDA-activated currents in cultured mouse cortical and hippocampal neurons. Brain Res. 738, 249–256 10.1016/S0006-8993(96)00780-9 8955520

[B82] Zimatkin S. M. Buben A. L. (2007). Ethanol oxidation in the living brain. Alcohol Alcohol. 42, 529–532 10.1093/alcalc/agm059 17660523

[B83] Zimatkin S. M. Pronko S. P. Vasiliou V. Gonzalez F. J. Deitrich R. A. (2006). Enzymatic mechanisms of ethanol oxidation in the brain. Alcohol. Clin. Exp. Res. 30, 1500–1505 10.1111/j.1530-0277.2006.00181.x 16930212

[B84] Zintzaras E. Stefanidis I. Santos M. Vidal F. (2006). Do alcohol-metabolizing enzyme gene polymorphisms increase the risk of alcoholism and alcoholic liver disease? Hepatology 43, 352–361 10.1002/hep.21023 16440362

